# Oviposition-Stimulant and Ovicidal Activities of *Moringa oleifera* Lectin on *Aedes aegypti*

**DOI:** 10.1371/journal.pone.0044840

**Published:** 2012-09-06

**Authors:** Nataly Diniz de Lima Santos, Kézia Santana de Moura, Thiago Henrique Napoleão, Geanne Karla Novais Santos, Luana Cassandra Breitenbach Barroso Coelho, Daniela Maria do Amaral Ferraz Navarro, Patrícia Maria Guedes Paiva

**Affiliations:** 1 Departamento de Bioquímica, Centro de Ciências Biológicas, Universidade Federal de Pernambuco, Cidade Universitária, Recife, Pernambuco, Brazil; 2 Departamento de Química Fundamental, Centro de Ciências Exatas e da Natureza, Universidade Federal de Pernambuco, Cidade Universitária, Recife, Pernambuco, Brazil; New Mexico State University, United States of America

## Abstract

**Background:**

Natural insecticides against the vector mosquito *Aedes aegypti* have been the object of research due to their high level of eco-safety. The water-soluble *Moringa oleifera* lectin (WSMoL) is a larvicidal agent against *A. aegypti*. This work reports the effects of WSMoL on oviposition and egg hatching of *A. aegypti*.

**Methodology/Principal Findings:**

WSMoL crude preparations (seed extract and 0–60 protein fraction), at 0.1 mg/mL protein concentration, did not affect oviposition, while *A. aegypti* gravid females laid their eggs preferentially (73%) in vessels containing isolated WSMoL (0.1 mg/mL), compared with vessels containing only distilled water (control). Volatile compounds were not detected in WSMoL preparation. The hatchability of fresh eggs deposited in the solutions in the oviposition assay was evaluated. The numbers of hatched larvae in seed extract, 0–60 protein fraction and WSMoL were 45±8.7 %, 20±11 % and 55±7.5 %, respectively, significantly (p<0.05) lower than in controls containing only distilled water (75–95%). Embryos were visualized inside fresh control eggs, but not within eggs that were laid and maintained in WSMoL solution. Ovicidal activity was also assessed using stored *A. aegypti* eggs. The protein concentrations able to reduce the hatching rate by 50% (EC50) were 0.32, 0.16 and 0.1 mg/mL for seed extract, 0–60 protein fraction and WSMoL, respectively. The absence of hatching of stored eggs treated with WSMoL at 0.3 mg/mL (EC99) after transfer to medium without lectin indicates that embryos within the eggs were killed by WSMoL. The reduction in hatching rate of *A. aegypti* was not linked to decrease in bacteria population.

**Conclusions/Significance:**

WSMoL acted both as a chemical stimulant cue for ovipositing females and ovicidal agent at a given concentration. The oviposition-stimulant and ovicidal activities, combined with the previously reported larvicidal activity, make WSMoL a very interesting candidate in integrated *A. aegypti* control.

## Introduction

*Aedes aegypti* Linnaeus is a domestic, daytime mosquito that breeds preferably in water storage containers, discarded plastic vials, and automobile tyres [1]. This mosquito has great importance in tropical and subtropical countries, since it is the vector of yellow and dengue fevers. Currently, dengue is the vector-borne disease that spreads most rapidly worldwide, with over two-fifths of the world's population at risk of infection [2], [3]. The absence of an effective vaccine makes the control of vector population the only way to minimize dengue spreading.

Worldwide, synthetic chemicals, mainly pyrethroids, carbamates and organophosphates are used to control adults, larvae, pupae and eggs of *A. aegypti*. These compounds pose high environmental risks, due to adverse effects on human and non-target organisms [4], [5]. In addition, their widespread usage has lead to mosquito resistance, compromising the effectiveness of control strategies [6]. It was also demonstrated that the presence of residual herbicides in ecosystems can reduce the sensitiveness of mosquito larvae to insecticides [7]. Due to their biodegradability, insecticides extracted from plants have been considered environmentally friendly substitutes for synthetic insecticides [8].

Plant extracts, secondary metabolites, essential oils and lectins (carbohydrate-binding and hemagglutinating proteins) have been shown to exert deleterious effect on *A. aegypti*, delaying development, impairing growth and digestive enzyme activities, reducing egg hatching and larval survival, as well as deterring feeding and oviposition activities [9]–[15]. The deleterious effects of lectins on insects have been associated with interaction of lectin with *N*-acetylglucosamine residues of chitin, a structural component in insects being found in peritrophic matrices, epidermal cuticles and tracheas [16].

Oviposition behavior of mosquitoes is a useful tool in determining female preference for oviposition sites. The acceptance or rejection by gravid females of a site for oviposition can be related to chemosensory cues (semiochemicals) which the insects detect through specialized cuticular structures, sensilla and chemosensory neurons in antennae, mouthparts, wing margins and legs [17]. Olfactory sensilla detect airborne volatiles, while gustatory sensilla respond to the treatment with chemicals of low-volatility [17].

Oviposition repellents have been the object of research designed to discover ways to eliminate oviposition sites and ward off *A. aegypti* gravid females from human hosts. Monoterpenes and sesquiterpenes present in essential oils act as repellant to females [18], [19]. Compounds that elicit increased *A. aegypti* oviposition have also been searched, an attractive property that may be used in ovitraps in combination with larvicidal agents [20]. Secondary metabolites produced by fungus *Trichoderma viride* and infusions obtained by fermentation of organic materials (e.g. sod, hay, grass, leaves, senescent bamboo and pelletized plant-based animal feeds) were shown to act as oviposition stimulants to gravid *Aedes* and *Culex* mosquitoes [21]–[24].

*A. aegypti* eggs are the main dispersion form of mosquito. Eggs resist dry conditions and manage to survive for many months or years in adverse environments [25]. They also afford these insects to rapidly reconstitute populations, even if the other stages have been eliminated by control measures. Thus, it is important to search for insecticides that act also against this developmental stage of the mosquito. Different plant species have been investigated for ovicidal activity on *A. aegypti*. Organic solvent extracts, as well as essential oils have been found to impair egg hatching [14], [26], [27].

*Moringa oleifera* Lamarck is a tree widely cultivated throughout the tropics and subtropics. In developing countries, its seeds are used to treat water for human consumption. Its seeds also contain a water-soluble lectin (WSMoL), which showed similarity with M02.1 and M02.2 (identification number gi|127215) proteins [9]. Using cell-free plasmid DNA and *Salmonella typhimurium* assays, a study on genotoxicity of WSMoL demonstrated that the lectin was non-mutagenic, indicating its safety for use in water treatment [28]. This lectin also acted as coagulant and antibacterial against *Staphylococcus aureus*, and effectively reduced lake water bacteria growth [29]. WSMoL exerted larvicidal activity (LC_50_ of 0.197 mg/mL) against *A. aegypti* fourth-stage larvae. The larvae treated with WSMoL lost the underlying epithelium, and showed increased gut lumen and hypertrophic segments [9]. Due to the high solubility in water and larvicidal activity against *A. aegypti*, WSMoL may become a potential candidate in *A. aegypti* control.

This work evaluated the effects of crude preparations (seed extract and 0–60 protein fraction) containing WSMoL and isolated lectin on *A. aegypti* oviposition. The hatchability of the freshly laid eggs obtained from the oviposition assay as well as of stored eggs was assessed. The presence of volatiles in WSMoL solution and the possibility that the effect of WSMoL on hatching involved embryo death and reduction of bacteria population in incubation medium were investigated. In addition, fresh and stored eggs from control and WSMoL treatments were examined on a stereomicroscope.

## Methods

### Breeding and rearing of A. aegypti in laboratory

The mosquitoes and eggs used were obtained from the colony (Rockfeller strain) maintained at the *Laboratório de Ecologia Química* of the *Universidade Federal de Pernambuco* (Recife, Brazil) since 2003. The insectary room was kept at 27±1°C, 78±2% relative humidity and 14∶10 (light/dark) photoperiod. The larvae were reared in plastic bowls containing water and cat food (Whiskas®). Adult mosquitoes were reared in cages (30×30×30 cm) covered with a fine mesh cloth and fed a 10% glucose solution. Females took blood meal from chicken blood acquired from local farms and dispensed from a common artificial feeder. The eggs laid by females were collected and used to restart the cycle for maintaining the colony or used to assess ovicidal activity.

### Crude preparations of WSMoL

*M. oleifera* (Family Moringaceae) has the vernacular names ‘‘moringa” in Portuguese, ‘‘árbol del ben” in Spanish, and horseradish tree or drumstick in English. Seeds were collected from 10–15-year-old trees in Recife City, State of Pernambuco, northeastern Brazil, and stored at −20°C. Voucher specimen (number 73,345) is archived at the herbarium Dárdano de Andrade Lima (Instituto Agronômico de Pernambuco, Recife, Brazil).

Crude preparations of WSMoL were obtained according to Coelho et al. [9]. Seeds were milled to a fine powder and homogenized (10 g) with distilled water (100 mL) using a magnetic stirrer for 16 h at 4°C. After filtration through gauze and centrifugation (9,000 g, 15 min, 4°C), the clear supernatant (seed extract) was submitted to protein precipitation using ammonium sulphate at saturation of 60% according to Green and Hughes [30]. The 0–60 precipitate collected after centrifugation (9,000 *g*, 15 min, 4°C) was dialyzed (3.5 kDa cut-off membrane) against distilled water (4 h) for use in bioassays and additionally with 0.15 M NaCl (4 h) for use in chromatography step. The dialysed fraction corresponded to the 0–60 protein fraction. The presence of residual ammonium sulphate in the 0–60 protein fraction was evaluated adding 50 µL of this sample to 0.5 mL of a 10 mg/mL barium chloride solution acidified to pH 4.0 with 1.0 M HCl. The formation of precipitates indicates that there is ammonium sulphate in sample [31].

### Isolation of WSMoL

WSMoL was isolated according to the procedure described by Coelho et al. [9]. The 0–60 protein fraction was loaded (40 mg of proteins) onto a chitin column (7.5×1.5 cm) equilibrated (0.3 mL/min flow rate) with 0.15 M NaCl, a salt concentration which does not interfere on *A. aegypti* oviposition [32]. After washing with the equilibrating solution, WSMoL was recovered by elution with 1.0 M acetic acid and dialysed (3.5 kDa cut-off membrane) against distilled water (4 h) at 4°C for eluent elimination. The presence of residual sodium chloride in WSMoL was evaluated according to the classic Mohr's method for determination of chloride by precipitation titration with silver nitrate. The detection limit of the method was 5×10^−4^ M.

### Protein and carbohydrate contents

The protein concentration was determined according to Lowry et al. [33] using bovine serum albumin (31.25–500 µg/mL) as standard. Carbohydrate concentration was determined according to Dubois et al. [34] using mannose (10–500 µg/mL) as standard.

### Hemagglutinating activity

Hemagglutinating activity of seed extract, 0–60 protein fraction and erythrocytes treated with glutaraldehyde [36]. One hemagglutination unit (titer) was defined as the reciprocal of the highest dilution of the sample promoting full agglutination of erythrocytes [11]. Specific hemagglutinating activity (unit WSMoL was assessed aiming to quantify and monitor lectin activity. The assay was performed in microtiter plates (Kartell S.P.A., Italy) according to Paiva and Coelho [35] using suspension (2.5% v/v) of rabbit /mg) was defined as the ratio between the titer and protein concentration.

### Oviposition assay

**Figure 1:**
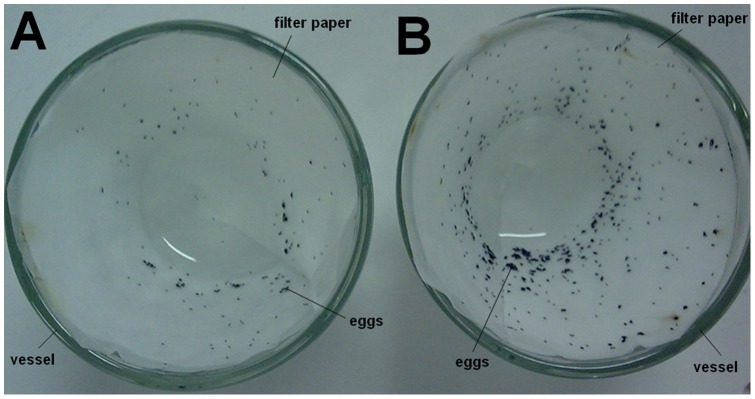
Top view of glass vessels used in oviposition assay. A filter paper cone was placed covering the inner of each vessel to provide a support for oviposition. Next, the vessels were filled with distilled water (A, control) or WSMoL at 0.1 mg/mL in distilled water (B).

Oviposition assay was performed according to Navarro et al. [32]. Oviposition assays were carried out using *A. aegypti* gravid females 3 days after blood meal. Twenty-five females were placed in a bioassay cage (33×21×30 cm) containing two glass vessels (10 cm diameter), each containing 50 mL of distilled water and placed at diagonally opposite corners of the cage. Aliquot (1 mL, 5.1 mg/mL of protein) of seed extract, 0–60 protein fraction or WSMoL was added to a vessel, resulting in a final protein concentration of 0.1 mg/mL. The same volume of distilled water was added to the control vessel. A disk shaped piece of filter paper (18 cm in diameter) folded into a cone was placed covering the inside of each vessel, to provide a support for oviposition (Figure 1). The females were maintained at 27±0.5°C with 73±0.4% relative humidity for 14 h in the dark. After this period, eggs in oviposition paper sheets were manually counted using a stereomicroscope (Leica M80). The oviposition response was expressed as follows: % oviposition  = 100× [(number of eggs in sample vessel) / (number of eggs in sample and control vessels)]. For each treatment (seed extract, 0–60 protein fraction or WSMoL), three independent experiments were performed in quintuplicate, on different dates.

### Volatiles analysis by headspace analysis

WSMoL preparation was enclosed within a glass vessel from which the air was drawn for 30 min by a battery-operated membrane pump (ASF Thomas) at a constant flow rate of 200 mL/min through sorbent traps containing a mixture of 0.05 g of Tenax TA (80/100 mesh, Macherey-Nagel 706318) and 0.05 g of Carbopack X (20/40 mesh, Supleco 1–0435). Blanks corresponded to the air drawn from empty vessel. The traps were eluted with 150 µL acetone, which was kept under −24°C refrigeration until analysis.

The presence of trapped volatiles was analyzed by combined gas-chromatography-mass spectrometry (GC-MS) on a Thermo Finnigan Voyager Mass Spectrometer coupled with a Thermo Trace GC 2000 (Thermo Fisher Scientific) equipped with a CP-Wax 52CB column (Varian; 30 m×0.25 mm×0.25 µm). The sample eluted from traps (1.0 µL) was injected in the column in splitless mode, and the temperature of the inlet was 250°C. GC oven temperature was set at 60°C for 3 min, increased by 2.5°C/min to 240°C, and then held steady for 10 min. Helium carrier gas flow was maintained at a constant pressure of 100 kPa. The MS interface was 200°C, and mass spectra were taken at 70 eV in EI mode) with a scanning speed of 0.5 scan/s from m/z 20–350.

### Hatchability of fresh eggs laid on seed extract, 0–60 protein fraction and WSMoL

Aiming to determine if *M. oleifera* seed preparations affect hatchability, fifty fresh eggs from each vessel from oviposition assays (including controls) were selected considering their integrity using a stereomicroscope and placed again in the same solutions where they were deposited. The number of hatched larvae present in solution was determined after 144 h of incubation at 28°C. For each treatment (seed extract, 0–60 protein fraction or WSMoL), three independent experiments were performed in quintuplicate on different dates.

### Ovicidal assay using stored eggs

Ovicidal assay was performed according to Prajapati et al. [37]. A. aegypti eggs stored for 3 months at 28°C were selected considering their integrity using a stereomicroscope. Seed extract was diluted in filtered tap water to provide test solutions with protein concentrations of 0.2, 0.3, 0.4, 0.5, 0.6, 0.7, 0.8, and 1.0 mg/mL. The same procedure was performed to provide test solutions of 0–60 protein fraction (0.1, 0.2, 0.3, 0.4, 0.5, and 0.6 mg/mL of protein) and purified WSMoL (0.03, 0.05, 0.08, 0.1, 0.13, and 0.15 mg/mL). The final volume of each ovicidal assay was 20 mL of test solution and contained 50–60 eggs. Controls contained distilled water, in a volume equivalent to that of sample used to achieve each concentration, completed to 20 mL with filtered tap water. The number of hatched larvae was determined after 72 h of incubation at 28°C. Three independent experiments were run in triplicate.

Aiming to evaluate whether WSMoL inhibits hatching and/or kills embryos, stored eggs submerged for 72 h in a 0.3 mg/mL lectin solution were rinsed with distilled water and transferred to another vessel filled with filtered tap water. The hatching of larvae was assessed after 24 and 48 h.

### Evaluation of bacteria population in ovicidal assays medium

Fresh and stored eggs were incubated with WSMoL at 0.1 and 0.3 mg/mL, respectively. After 144 (fresh eggs) or 72 h (stored eggs), the media (50 µL) from treatments or controls were smeared on petri dishes containing nutrient agar. The plates were incubated at 37°C for 16 h. After, the number of bacterial colony forming units (CFU) was determined.

### Visualization of embryos in fresh and stored eggs from ovicidal assays

Fresh and stored eggs were incubated with WSMoL at 0.1 and 0.3 mg/mL, respectively. After 144 h (fresh) or 72 h (stored), the eggs were placed in solution of 5% sodium hypochlorite until all the chorion had dissolved, leaving the vitelline membrane intact [38]. The eggs were then rinsed with distilled water to prevent complete dissolution by sodium hypoclorite and visualized in a Leica KL300 stereomicroscope (Leica Microsystems, Wetzlar, Germany). Fresh eggs incubated with water and stored eggs not incubated with any solution were also observed.

### Statistical analysis

Standard deviations (s.d.) were calculated using GraphPad Prism version 4.0 for Windows (GraphPad Software, San Diego, California, USA) and data were expressed as a mean of replicates ± s.d. Significant differences between treatment groups were analysed by Student's *t*-test (significance at p<0.05) using Origin 6.0 program. The effective concentrations required to reduce the hatching of *A. aegypti* eggs by 50% and 99% (EC_50_ and EC_99_) in 72 h were calculated by probit analysis with a reliability interval of 95% (degrees of freedom of 5, 4 and 4 for tests with seed extract, 0–60 protein fraction and WSMoL, respectively) using the computer software IBM SPSS Statistics (IBM Corporation, New York, USA).

## Results

In seed extract, protein concentration was 4.1 mg/mL and specific hemagglutinating activity was 15. In 0–60 protein fraction, protein concentration was 21.0 mg/mL and specific hemagglutinating activity was 97. No precipitate formation was observed after addition of 0–60 protein fraction to acidified barium chloride solution, revealing that no residual ammonium sulphate remained in the sample. WSMoL was isolated by chitin chromatography and was the most hemagglutinating preparation (specific hemagglutinating activity of 2,915). The results from Mohr's method did not reveal the presence of chloride ions in solution, indicating absence of residual sodium chloride content. Carbohydrate contents in seed extract, 0–60 protein fraction were 0.2 and 0.008 mg/mL, respectively and no detected in WSMoL.

**Figure 2:**
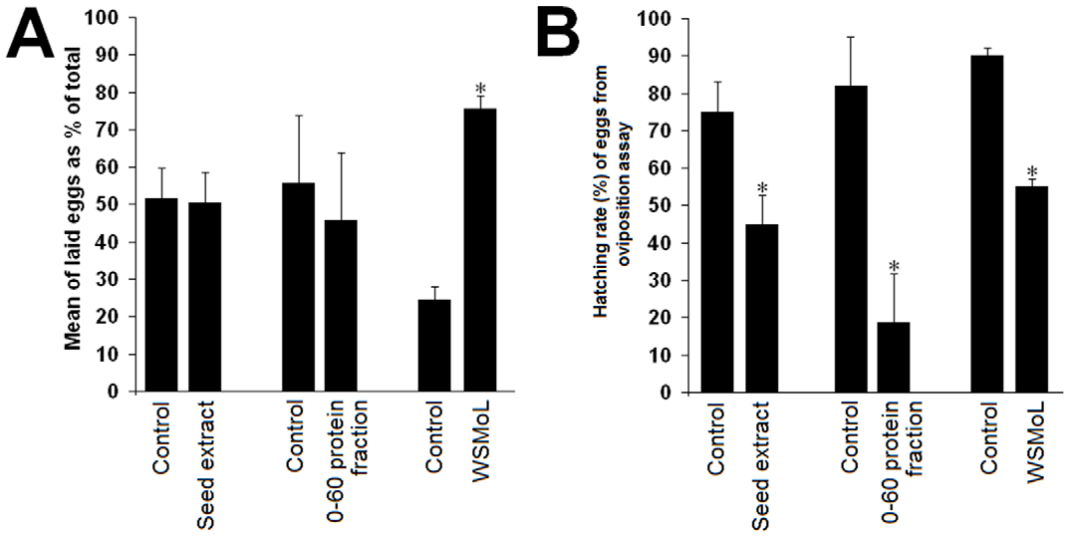
Effect of *M. oleifera* seed preparations (0.1 mg/mL of protein) on oviposition and egg hatching of *Aedes aegypti*. (A) Mean of eggs laid by *A. aegypti* gravid females in distilled water and *M. oleifera* seed preparations. The oviposition response was evaluated by double-choice bioassays. Three distinct assays (“control vs. seed extract”, “control vs. 0–60 protein fraction” and “control vs. WSMoL”) were performed separately, each one with its respective control. The oviposition response was expressed as: % oviposition  = 100× [(number of eggs in sample vessel) / (number of eggs in sample and control vessels)]. (B) Hatching rate (%) of eggs laid by gravid females during the oviposition assays in distilled water and *M. oleifera* seed preparations. (*) indicates significant differences (p<0.05) between control and test groups.

The effects of *M. oleifera* seed preparations on *A. aegypti* oviposition are shown in Fig. 2A. The difference between the amount of eggs deposited in seed extract and control vessels was not significant (p>0.05), showing that this preparation was not able to attract or repel *A. aegypti* females. Similarly to the results found for seed extract, *A. aegypti* gravid females did not show (p>0.05) preference or rejection for vessels containing the 0–60 protein fraction. The number of eggs laid in vessels containing WSMoL was significantly (p<0.05) higher than that laid in control vessels, showing that the lectin exerts an oviposition-stimulant activity.

Volatile compounds were not detected in WSMoL preparation by dynamic headspace analysis since chromatograms from GC-MS did not reveal the presence of any such compound.

The effect of *M. oleifera* preparations on *A. aegypti* eggs from oviposition assay (fresh eggs) was evaluated aiming at determining if the hatchability of eggs laid on lectin solution would be affected. Fig. 2B shows that hatching rates of fresh eggs from seed extract, 0–60 protein fraction and WSMoL assays at concentration of 0.1 mg/mL were 45%, 20% and 55%, respectively. Significant reductions (p<0.05) in hatching rate of fresh eggs were observed after 144-h incubation with seed extract, 0–60 protein fraction and WSMoL in comparison with their respective controls.

**Figure 3:**
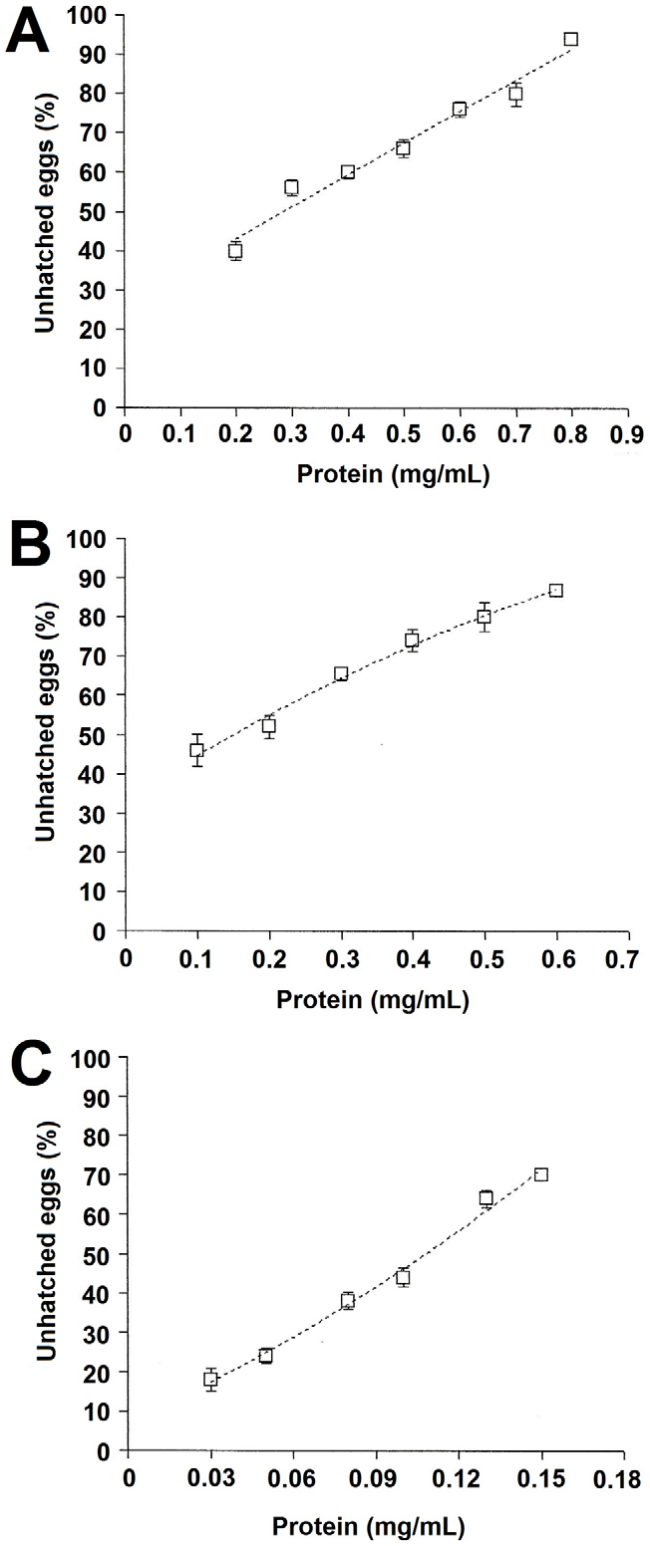
Percentage of unhatched stored eggs after incubation with seed extract (A), 0–60 protein fraction (B) and WSMoL (C) for 72 h.

The hatching rate of stored eggs was also reduced after treatment with seed extract, 0–60 protein fraction and WSMoL in a dose-dependent manner (Fig. 3). The values of EC_50_ are shown in Table 1. When eggs submerged for 72 h in WSMoL at EC99 (0.3 mg/mL) were transferred to medium containing only tap water, the hatching rate was zero after 24 and 48 h. The absence of dead larvae in WSMoL solutions at 0.03, 0.05, 0.08, 0.1, 0.13, 0.15 mg/mL and no hatching of eggs treated with WSMoL at EC_99_ reflect the ovicidal, not the larvicidal activity of lectin.

**Table 1:** Ovicidal activity on *A. aegypti* stored eggs of *M. oleifera* seed extract, 0–60 protein fraction and WSMoL.

Sample	EC_50_ (mg/mL) a	EC_99_ a
Seed extract	0.32 [0.25–0.37]	1.18 [1.05–1.38]
0–60 protein fraction	0.16 [0.1–0.22]	1.14 [0.96–1.44]
WSMoL	0.10 [0.09–0.12]	0.30 [0.25–0.38]

a Effective concentrations of proteins required to reduce in 50% (EC_50_) and 99% (EC_99_) the hatching of *A. aegypti* eggs in 72 h calculated by probit analysis with a reliability interval of 95%. Values in square brackets represent the lower and upper endpoints for reliability interval.

The numbers of CFU from ovicidal assays using fresh and stored eggs incubated with WSMoL were 1.4×103±28 and 1.3×103±73 CFU/mL, respectively. Media from controls of assays with fresh and stored eggs presented 1.5×103±135 and 1.3×103±94 CFU/mL, respectively.

**Figure 4:**
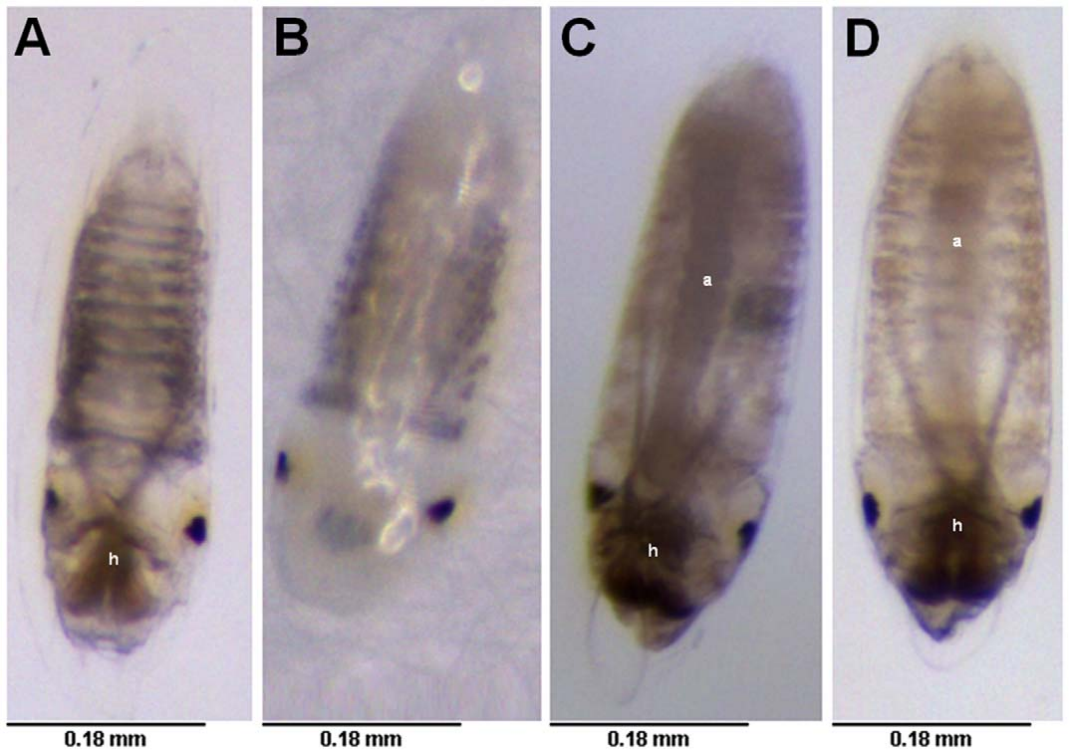
Visualization of *A. aegypti* fresh and stored eggs exposed or not to WSMoL in stereomicroscope (x80) after clearing of the heavily pigmented chorion with 5% sodium hypochlorite. (A) Fresh egg laid and maintained in distilled water (control). (B) Fresh egg laid and maintained in WSMoL (0.1 mg/mL). (C) Stored egg which was not treated with any solution. (D) Stored egg that did not hatch after incubation with WSMoL at EC_99_ (0.3 mg/mL) for 72 h. (h) head; (a) abdomen.

The visualization on stereomicroscope of fresh eggs deposited in distilled water (control) or WSMoL (0.1 mg/mL) and maintained for 72 h in these same solutions revealed the presence of embryo head inside eggs from control treatment (Figure 4A), but the embryo could not be visualized through the vitelline membrane in eggs from WSMoL treatment (Figure 4B). Embryos were visualized in stored eggs incubated with WSMoL for 72 h and from control (Figure 4C and 4D).

## Discussion

The results obtained for WSMoL isolation were similar to those obtained by the protocol previously described by Coelho et al. [9]. The lower values of specific hemagglutinating activity in seed extract and 0–60 protein fraction, in comparison with isolated lectin, indicate the lowest concentration of WSMoL. The measurement of the biological activity of a protein is essential to assure that it is active before determination of a biological activity. In lectinology, the hemagglutinating assay is the classic tool to assess the carbohydrate-binding property of a lectin, making sure that it is active. Increased specific hemagglutinating activity reveals lectin concentration and purification [39].

Ammonium sulphate precipitation is a rapid and inexpensive method broadly used to concentrate proteins. This salt does not affect structure and function, and can be easily removed from the protein solution by exhaustive dialysis [9]–[11], [30], [34], [40]. The absence of precipitate species after treatment of 0–60 protein fraction with acidified barium chloride solution proved the absence of residual ammonium sulphate in this preparation.

The results from oviposition assays revealed that WSMoL at 0.1 mg/mL with specific hemagglutinating of 2,915 acted as a chemical cue for *A. aegypti* gravid females, eliciting increased oviposition response. The absence of oviposition-stimulant activity in seed extract and 0–60 protein fraction was probably due to the lowest specific hemagglutinating activity of these preparations. To the best of our knowledge, there are no reports of oviposition deterrent, attractive or stimulant response from mosquitoes induced by plant lectins. The 0.1 mg/mL (or 100 ppm) concentration was selected, since this value is often used in oviposition bioassays [18], [41], [42].

Prabhu et al. [43] reported that methanolic extract from *M. oleifera* seeds (0.5, 1.0 and 2.0 mg/cm2) repelled *Anopheles stephensi*. The authors observed a decrease in number of bites in arms of human volunteers treated with the extract. The fact that the seed extract used here did not exert repellent activity reveals that repellent compounds were not extracted in water or that, if present at all, concentrations were below the detection threshold by females.

Oviposition-stimulant activity of WSMoL can be due to gustatory stimuli, since non-volatile chemicals such as proteins are perceived by gustatory system of insects [44]. The stimulus in oviposition can be unchained by WSMoL adsorption onto gustatory sensillas of *A. aegypti* female, as reported in a study on the detection of sex pheromones and protein kairomones by *Glossina* spp. and *Diadromus pulchellus*, respectively [45], [46]. Volatile compounds were not detected by dynamic headspace analysis in WSMoL preparation indicating that, if present, they occurred in trace amounts. Olfactory stimuli by volatile compounds were described for selection of oviposition sites by *Culex quinquefasciatus, A. aegypti* and *Aedes triseriatus* [47].

WSMoL from chitin column was dialysed against distilled water aiming to eliminate acetic acid used in elution as well as residues of 0.15 M NaCl (0.85%) used in washing steps of chromatography. TianFu et al. [48] reported that acetic acid (0.001 mg/L) acted as an oviposition attractant to *Aedes albopictus* gravid females. In the present study, GC-MS analysis did not identify acetic acid in WSMoL. Thus, the effect of lectin preparation was not due to contamination by this compound. Chloride ions were not detected by Mohr's method in WSMoL, indicating that the oviposition-stimulant effect of lectin cannot be linked to salt contamination. Also, Navarro et al. [32] demonstrated that NaCl at 0 to 5% concentrations did not interfere on *A. aegypti* oviposition.

Frings and Hamrum [49] reported that sugars can be detected through contact receptors present in labella of *A. aegypti* mosquito. Thus, *M. oleifera* preparations were evaluated for presence of these compounds. The results reveal that carbohydrates from *M. oleifera* seed preparations were not attractive components, since WSMoL, which showed the highest oviposition rate, was free of carbohydrates. WSMoL can be included in the group of non-volatile proteins with oviposition-stimulant activity on *A. aegypti*.

Since the embryogenesis of *A. aegypti* eggs is completed between 77–96 h and acquisition of egg resistance occurs 48 h after post-oviposition [25], [50] the effect on hatchability of eggs from oviposition assay was assessed. The hatching rates were determined after 144 h to guarantee that the eggs had a reasonable time to complete embryonic development. The results showed that besides stimulating oviposition by females, WSMoL at 0.1 mg/mL was also able to impair hatching, an advantage if it will be used in ovitraps.

The effect of WSMoL preparations on stored eggs was also evaluated during an incubation period of 72 h. Incubation periods between 72 and 120 h have been used to assess ovicidal activity on eggs which have already completed their embryogenesis [51]–[56]. The detection of lowest EC_50_ value for WSMoL suggests that the lectin is an active principle in seed extract and 0–60 protein fraction against stored eggs, although a proportional correlation between increase in specific hemagglutinating activity and ovicidal effect (decrease in EC_50_) was not observed. The absence of hatching of stored eggs treated with WSMoL at 0.3 mg/mL (EC_99_) after transfer to medium without lectin indicates that embryos within the eggs were killed by WSMoL. The absence of contaminant traces of acetic acid, sodium chloride and ammonium sulphate in 0–60 protein fraction and WSMoL assure that the adverse effects of these preparations on hatching rates were not due to residues of these chemicals.

It has been reported that the presence of bacteria is a hatching-stimulant factor for *A. aegypti* eggs and in the absence of bacteria the hatchability is lower than 5% [57]. Since it is known that WSMoL exerts antibacterial effect [29], it was evaluated whether the lower hatching rate could have been also a consequence of reduction in bacteria population. The numbers of CFU/mL in media from ovicidal assays using WSMoL was not lower than that detected in media from control assays. This result reveals that, in this case, lower hatching rates in WSMoL treatment were not linked to decrease in bacterial population. So, it is possible to conclude that impairment of egg hatching by WSMoL was due to embryo death.

It was also evaluated if the sensitiveness of freshly laid eggs to *M. oleifera* preparations was different from that of stored eggs. According to probit analysis of results from ovicidal assays using stored eggs, the egg hatching rates after treatment with seed extract, 0–60 protein fraction and WSMoL at concentration of 0.1 mg/mL would be 65%, 54% and 50%, respectively. Comparison of data from ovicidal assays using fresh and stored eggs and protein concentration of 0.1 mg/mL reveals that stored eggs were less sensitive to seed extract and 0–60 protein fraction, while WSMoL reduced similarly the hatching rates of fresh and stored eggs. These findings indicate the presence of other ovicidal agents in seed extract and 0–60 protein fraction that were eliminated in the WSMoL isolation procedure. Ferreira et al. [58] did not detect ovicidal activity from aqueous extract from dehulled *M. oleifera* seeds (5.2 mg/mL) using stored eggs. The authors attributed this fact to the high resistance of eggs. Differently, WSMoL showed similar ovicidal activity against fresh and stored eggs, indicating that the completion of embryogenesis and egg maturation processes was not accompanied by reduction in sensitiveness to lectin. This is an interesting and advantageous property concerning *A. aegypti* control strategies targeting the eggs, the most resistant stage of mosquito life cycle.

The EC_50_ of WSMoL on stored eggs was lower than values determined for essential oils from *Juniperus macropoda, Zingiber officinale* and *Pimpinella anisum*, whose EC_50_ ranged from 0.15 to 0.18 mg/mL and leaf extracts of *Eclipta alba*, whose EC_50_ ranged from 0.10 to 0.20 mg/mL [27], [37]. The ovicidal activity of WSMoL was compared with those from essential oils and plant extracts because there are no previous reports on ovicidal activity of lectins on insects.

Ovicidal compounds are able to interrupt embryo development, impair the survival of larva inside the egg or block egg hatching [27]. Fresh eggs from control treatment showed embryogenesis in progress while impairment of embryo development was detected in fresh eggs treated with WSMoL, reflecting the ovicidal activity.

Chitin is present in oocytes, eggshell and eggs of *A. aegypti* and this polysaccharide has an important role in egg viability [50]. The ovicidal activity of WSMoL may be linked to binding of lectin to chitin present in eggshells, blocking the hatching process by promoting disruption of this structure. Also, the fact that stored eggs transferred to water after incubation with WSMoL for 72 h did not hatch reveals that embryos, observed inside eggs using a stereomicroscope, are dead. It is possible that WSMoL gets inside the egg, interfering in embryo development through the same mechanisms which kill *A. aegypti* larvae.

The previously described larvicidal activity of WSMoL, combined with its oviposition-stimulant and ovicidal activities described here make this lectin a very interesting candidate for use in integrated mosquito control programs. The WSMoL concentration in which oviposition-stimulant (0.1 mg/mL) and ovicidal (EC_50_ of 0.1 mg/mL) activities were detected was lower than that in which the lectin promotes larvicidal activity (LC_50_ of 0.197 mg/mL). This finding is interesting, because if WSMoL is applied in an *A. aegypti* breeding site aiming at killing the larvae, it will also attract gravid females and will impair the hatching of new eggs laid by females.

In summary, WSMoL is a sustainable and environmentally friendly alternative for *A. aegypti* control, since one same concentration of WSMoL acts as a chemical stimulant cue for ovipositing females and ovicidal agent at the same time. Further studies will be performed on oviposition response of gravid females under field conditions as well as on the development of effective formulations for WSMoL and its large-scale production based on genetic engineering techniques.

## References

[1] World Health Organization (2009) Dengue and dengue haemorrhagic fever. Fact sheet N° 117. Available: http://www.who.int/mediacentre/factsheets/fs117.

[2] Murrell S, Wu S-C, Butler M (2011) Review of dengue virus and the development of a vaccine. Biotechnol Adv 29: 239–247. 21146601 10.1016/j.biotechadv.2010.11.008

[3] Urdaneta-Marquez L, Failloux A-B (2011) Population genetic structure of *Aedes aegypti*, the principal vector of dengue viruses. Infect Genet Evol 11: 253–261. 21167319 10.1016/j.meegid.2010.11.020

[4] Sánchez-Fortún S, Barahona MV (2005) Comparative study on the environmental risk induced by several pyrethroids in estuarine and freshwater invertebrate organisms. Chemosphere 59: 553–559. 15788178 10.1016/j.chemosphere.2004.12.023

[5] Rodriguez MM, Bisset JA, Fernández D (2007) Levels of insecticide resistance and resistance mechanisms in *Aedes aegypti* from some Latin American countries. J Am Mosq Control Assoc 23, 420–429. 10.2987/5588.1 18240518

[6] Perry T, Batterham P, Daborn PJ (2011) The biology of insecticidal activity and resistance. Insect Biochem Mol Biol 41: 411–422. 21426939 10.1016/j.ibmb.2011.03.003

[7] Boyer S, Sérandour J, Lempérière G, Raveton M, Ravanel P (2006) Do herbicide treatments reduce the sensitivity of mosquito larvae to insecticides? Chemosphere 65: 721–724. 16574189 10.1016/j.chemosphere.2006.02.032

[8] Regnault-Roger C, Staff V, Philogène B, Terrón P, Vincent C (2004) Biopesticidas de origen vegetal, Madrid: Ediciones Mundi-Prensa.

[9] Coelho JS, Santos NDL, Napoleão TH, Gomes FS, Ferreira RS, et al (2009) Effect of *Moringa oleifera* lectin on development and mortality of *Aedes aegypti* larvae. Chemosphere 77: 934–938. 19747711 10.1016/j.chemosphere.2009.08.022

[10] Sá RA, Santos NDL, Silva CSB, Napoleão TH, Gomes FS, et al (2009) Larvicidal activity of lectins from *Myracrodruon urundeuva* on *Aedes aegypti* . Comp Biochem Physiol C 149: 300–306. 10.1016/j.cbpc.2008.08.004 18761426

[11] Napoleão TH, Pontual EV, Lima TA, Santos NDL, Sá RA, et al (2012) Effect of *Myracrodruon urundeuva* leaf lectin on survival and digestive enzymes of *Aedes aegypti* larvae. Parasitol Res 110: 609–616. 21735148 10.1007/s00436-011-2529-7

[12] Mandal S (2010) Exploration of larvicidal and adult emergence inhibition activities of *Ricinus communis* seed extract against three potential mosquito vectors in Kolkata, India. Asian Pacific J Trop Med 3: 605–609.

[13] Santos SRL, Melo MA, Cardoso AV, Santos RLC, de Sousa DP, et al (2011) Structure-activity relationships of larvicidal monoterpenes and derivatives against *Aedes aegypti* Linn. Chemosphere 84: 150–153. 21376365 10.1016/j.chemosphere.2011.02.018

[14] Pontual EV, Napoleão TH, de Assis CRD, Bezerra RS, Xavier HS, et al (2012) Effect of *Moringa oleifera* flower extract on larval trypsin and acethylcholinesterase activities in *Aedes aegypti* . Arch Insect Biochem Physiol 19: 135–152. 10.1002/arch.21012 22392801

[15] Warikoo R, Wahab N, Kumar S (2011) Oviposition-altering and ovicidal potentials of five essential oils against female adults of the dengue vector, *Aedes aegypti* L. Parasitol Res. 109: 1125–1131. 10.1007/s00436-011-2355-y 21445613

[16] Merzendorfer H, Zimoch L (2003) Chitin metabolism in insects: structure, function and regulation of chitin synthases and chitinases. J Exp Biol 206: 4393–4412. 14610026 10.1242/jeb.00709

[17] Bohbot J, Vogt RG (2005) Antennal expressed genes of the yellow fever mosquito (*Aedes aegypti* L.); characterization of odorant-binding protein 10 and takeout. Insect Biochem Mol Biol 35: 961–979. 15978998 10.1016/j.ibmb.2005.03.010

[18] Autran ES, Neves IA, da Silva CSB, Santos GKN, da Câmara CA, et al (2009) Chemical composition, oviposition deterrent and larvicidal activities against *Aedes aegypti* of essential oils from *Piper marginatum* Jacq. (Piperaceae). Bioresour Technol 100: 2284–2288. 19070480 10.1016/j.biortech.2008.10.055

[19] Nerio LS, Olivero-Verbel J, Stashenko E (2010) Repellent activity of essential oils: A review. Bioresour Technol 101: 372–378. 19729299 10.1016/j.biortech.2009.07.048

[20] Barbosa RMR, Regis LN (2011) Monitoring temporal fluctuations of *Culex quinquefasciatus* using oviposition traps containing attractant and larvicide in an urban environment in Recife, Brazil. Mem Inst Oswaldo Cruz 106: 451–455. 21739033 10.1590/s0074-02762011000400011

[21] Lampman RL, Novak RJ (1996) Attraction of *Aedes albopictus* adults to sod infusion. J Am Mosq Control Assoc 12: 119–124. 8723268

[22] Ritchie SA (2001) Effect of some animal feeds and oviposition substrates on *Aedes* oviposition in ovitraps in Cairns, Australia. J Am Mosq Control Assoc 17: 206–208. 14529089

[23] Ponnusamy L, Xu N, Böröczky K, Wesson DM, Ayyash LA, et al (2010) Oviposition responses of the mosquitoes *Aedes aegypti* and *Aedes albopictus* to experimental plant infusions in laboratory bioassays. J Chem Ecol 36: 709–719. 20521087 10.1007/s10886-010-9806-2 PMC4562425

[24] Santos E, Correia J, Muniz L, Meiado M, Albuquerque C (2010) Oviposition activity of *Aedes aegypti* L. (Diptera: Culicidae) in response to different organic infusions. Neotrop Entomol 39: 299–302. 20498970 10.1590/s1519-566x2010000200023

[25] Russell BM, Kay BH, Shipton W (2001) Survival of *Aedes aegypti* (Diptera: Culicidae) eggs in surface and subterranean breeding sites during the Northern Queensland dry season. J Med Entomol 38: 441–445. 11372971 10.1603/0022-2585-38.3.441

[26] Govindarajan M (2011) Evaluation of *Andrographis paniculata* Burm.f. (Family: Acanthaceae) extracts against *Culex quinquefasciatus* (Say.) and *Aedes aegypti* (Linn.) (Diptera:Culicidae). Asian Pacific J Trop Med 4: 176–181. 10.1016/S1995-7645(11)60064-3 21771448

[27] Govindarajan M, Karuppannan P (2011) Mosquito larvicidal and ovicidal properties of *Eclipta alba* (L.) Hassk (Asteraceae) against chikungunya vector, *Aedes aegypti* (Linn.) (Diptera: Culicidae). Asian Pacific J Trop Med 4: 24–28. 10.1016/S1995-7645(11)60026-6 21771410

[28] Rolim LADMM, Macêdo MFS, Sisenando HA, Napoleão TH, Felzenszwalb I, et al (2011) Genotoxicity evaluation of *Moringa oleifera* seed extract and lectin. J Food Sci 76: T53–T58. 21535795 10.1111/j.1750-3841.2010.01990.x

[29] Ferreira RS, Napoleão TH, Santos AFS, Sá RA, Carneiro-da-Cunha MG, et al (2011) Coagulant and antibacterial activities of the water-soluble seed lectin from *Moringa oleifera* . Lett Appl Microbiol 53: 186–192. 21605145 10.1111/j.1472-765X.2011.03089.x

[30] Green AA, Hughes L (1955) Protein fractionation on the basis of solubility in aqueous solution of salts and organic solvents. In: Colowick S, Kaplan N, editors. Methods in Enzymology, New York: Academic Press. 67–90.

[31] Hay FC, Westwood OMR (2002) Practical Immunology, 4^th^ ed, Oxford: Blackwell Science.

[32] Navarro DMAF, Oliveira PES, Potting RJP, Brito AC, Fital SJF, et al (2003) The potential attractant or repellent effects of different water types on oviposition in *Aedes aegypti* L (Dipt., Culicidae). J Appl Entomol 127: 46–50.

[33] Lowry OH, Rosebrough NJ, Farr AL, Randall RJ (1951) Protein measurement with the Folin phenol reagent. J Biol Chem 193: 265–275. 14907713

[34] Dubois M, Gilles KA, Hamilton JK, Rebers PA, Smith F (1956) Colorimetric method for determination of sugar and related substances. Anal Chem 28: 350–356.

[35] Paiva PMG, Coelho LCBB (1992) Purification and partial characterization of two lectin isoforms from *Cratylia mollis* Mart (camaratu bean). Appl Biochem Biotechnol 36: 113–118.

[36] Bing DH, Weyand JG, Stavinsky AB (1967) Hemagglutination with aldehyde fixed erythrocytes for assay of antigens and antibodies. Proc Soc Exp Biol Med 124: 1166–1170. 6024827 10.3181/00379727-124-31953

[37] Prajapati V, Tripathi AK, Aggarwal KK, Khanuja SPS (2005) Insecticidal, repellent and oviposition-deterrent activity of selected essential oils against *Anopheles stephensi, Aedes aegypti* and *Culex quinquefasciatus* . Bioresour Technol 96: 1749–1757. 16051081 10.1016/j.biortech.2005.01.007

[38] Mortenson EW (1950) The use of hypochlorite to study *Aedes nigromaculis* (Ludlow) embryos (Diptera: Culicidae). Mosq News 10: 211–212.

[39] Paiva PMG, Gomes FS, Napoleão TH, Sá RA, Correia MTS, et al.. (2010) Antimicrobial activity of secondary metabolites and lectins from plants. In: Méndez-Vilas A, editor. Current Research, Technology and Education Topics in Applied Microbiology and Microbial Biotechnology, Badajoz: Formatex. 396–406.

[40] Kent UM (1999) Purification of antibodies using ammonium sulfate fractionation or gel filtration. Meth Mol Biol 115: 11–18. 10.1385/1-59259-213-9:11 10098159

[41] Santos GKN, Dutra KA, Barros RA, da Câmara CAG, Lira DD, et al (2012) Essential oils from *Alpinia purpurata* (Zingiberaceae): Chemical composition, oviposition deterrence, larvicidal and antibacterial activity. Ind Crop Prod 40: 254–260.

[42] Consolo RAGB, Mendes NM, Pereira JP, Santos BS, Lamounier MA (1989) Influence of several plant extracts on the oviposition behaviour of *Aedes fluviatilis* (Lutz) (Diptera: Culicidae) in the laboratory. Mem Inst Oswaldo Cruz 84: 47–51.

[43] Prabhu K, Murugan K, Nareshkumar A, Ramasubramanian N, Bragadeeswaran S (2011) Larvicidal and repellent potential of *Moringa oleifera* against malarial vector, *Anopheles stephensi* Liston (Insecta: Diptera: Culicidae). Asian Pacific J Trop Biomed 1: 124–129. 10.1016/S2221-1691(11)60009-9 PMC3609168 23569741

[44] Beehler JW, Millar JG, Mulla MS (1994) Protein hydrolysates and associated bacterial contaminants as oviposition attractants for the mosquito *Culex quinquefasciatus* . Med Vet Entomol 8: 381–385. 7841493 10.1111/j.1365-2915.1994.tb00103.x

[45] Huyton PM, Langley PA, Carlson DA, Schwarz M (1980) Specificity of contact sex pheromones in tsetse flies, *Glossina* spp. Physiol Entomol 5: 253–264.

[46] Bénédet F, Leroy T, Gauthier N, Thibaudeau C, Thibout E, et al (2002) Gustatory sensilla sensitive to protein kairomones trigger host acceptance by an endoparasitoid. Proc R Soc Lond B 269: 1879–1886. 10.1098/rspb.2002.2077 PMC1691116 12350249

[47] Bentley MD, McDaniel IN, Yatagai M, Lee H-P, Maynard R (1979) p-Cresol: an oviposition attractant of *Aedes triseriatus* . Environ Entomol 8: 206–209.

[48] TianFu D, JianChu M, JianAn C (2009) Study on oviposition attractants to *Aedes albopictus* . J Zhejiang Univ (Agric Life Sci) 35: 532–536.

[49] Frings H, Hamrum CL (1950) The contact chemoreceptors of adult yellow fever mosquitoes, *Aedes aegypti* . J NY Entomol Soc 58: 133–142.

[50] Moreira MF, Santos AS, Marotta HR, Mansur JF, Ramos IB, et al (2007) A chitin-like component in *Aedes aegypti* eggshells, eggs and ovaries. Insect Biochem Mol Biol 37: 1249–1261. 17967344 10.1016/j.ibmb.2007.07.017

[51] Chenniappan K, Kadarkarai M (2008) Oviposition deterrent, ovicidal and gravid mortality effects of ethanolic extract of *Andrographis paniculata* Nees against the malarial vector *Anopheles stephensi* Liston(Diptera: Culicidae). Entomol Res 38: 119–125.

[52] Govindarajan M, Jebanesan A, Pushpanathan T (2008) Larvicidal and ovicidal activity of *Cassia fistula* Linn. Leaf extract against filarial and malarial vector mosquitoes. Parasitol Res 102: 289–292. 17989995 10.1007/s00436-007-0761-y

[53] Maheswaran R, Ignacimuthu S (2012) A novel herbal formulation against dengue vector mosquitoes *Aedes aegypti* and *Aedes albopictus* . Parasitol Res 110: 1801–1813. 22042505 10.1007/s00436-011-2702-z

[54] Elango G, Bagavan A, Kamaraj C, Zahir AA, Rahuman AA (2008) Oviposition-deterrent, ovicidal, and repellent activities of indigenous plant extracts against *Anopheles subpictus* Grassi (Diptera: Culicidae). Parasitol Res 103: 691–695. 19707789 10.1007/s00436-009-1593-8

[55] Samidurai K, Saravanakumar A (2011) Mosquitocidal properties of nereistoxin against *Anopheles stephensi*, *Aedes aegypti* and *Culex quinquefasciatus* (Diptera: Culicidae). Parasitol Res 109: 1107–1112. 21472401 10.1007/s00436-011-2353-0

[56] Ramos MV, Bandeira GP, Freitas CDT, Nogueira NAP, Alencar NMN, et al (2006) Latex constituents from *Calotropis procera* (R. Br.) display toxicity upon egg hatching and larvae of *Aedes aegypti* (Linn.). Mem Inst Oswaldo Cruz 101: 503–510. 17072453 10.1590/s0074-02762006000500004

[57] Ponnusamy L, Böröczky K, Wesson DM, Schal C, Apperson CS (2011) Bacteria stimulate hatching of yellow fever mosquito eggs. PLoS ONE 6: e24409. 21915323 10.1371/journal.pone.0024409 PMC3167859

[58] Ferreira PMP, Carvalho AFU, Farias DF, Cariolano NG, Melo VMM, et al (2009) Larvicidal activity of the water extract of *Moringa oleifera* seeds against *Aedes aegypti* and its toxicity upon laboratory animals. Anais Acad Brasil Ciên 81: 207–216. 10.1590/s0001-37652009000200007 19488625

